# HBV promotes its replication by up-regulating RAD51C gene expression

**DOI:** 10.1038/s41598-024-53047-7

**Published:** 2024-01-31

**Authors:** Ting-wei Peng, Qing-feng Ma, Jie Li, Xue Wang, Cong-hui Zhang, Junwen Ma, Jun-yi Li, Wei Wang, Cheng-liang Zhu, Xing-hui Liu

**Affiliations:** 1 Department of Clinical Laboratory, Shanghai Gongli Hospital, the Second Military Medical University, Shanghai, 200135 China; 2 Department of Clinical Laboratory, Liyuan Hospital of Tongji Medical College, Huazhong University of Science and Technology, Wuhan, 430077 China; 3 China Medical Tribune, Beijing, 100009 China; 4 Department of Clinical Laboratory, Wuhan Fourth Hospital, Wuhan, 430034 China; 5 Department of Clinical Laboratory, Renmin Hospital of Wuhan University, Wuhan, 430060 China

**Keywords:** Cancer, Molecular biology

## Abstract

Chronic hepatitis B virus (HBV) infection is a major cause of hepatocellular carcinoma (HCC), pegylated-interferon-α(PEG-IFNα) and long-term nucleos(t)ide analogs (NUCs) are mainly drugs used to treat HBV infection, but the effectiveness is unsatisfactory in different populations, the exploration of novel therapeutic approaches is necessary. RAD51C is associated with DNA damage repair and plays an important role in the development and progression of tumors. Early cDNA microarray results showed that RAD51C expression was significantly increased in HBV-infected HCC cells, however, the relationship between HBV infection and abnormal expression of RAD51C has not been reported. Therefore, we conducted RT-PCR, western blot, Co-immunoprecipitation(Co-IP), and immunofluorescence(IF) to detect HBV-RAD51C interaction in RAD51C overexpression or interfering HCC cells. Our results showed that RAD51C and HBV X protein(HBX) produced a direct interaction in the nucleus, the HBV infection of HCC cells promoted RAD51C expression, and the increased expression of RAD51C promoted HBV replication. This indicated that RAD51C is closely related to the occurrence and development of HCC caused by HBV infection, and may bring a breakthrough in the the prevention and treatment study of HCC.

## Introduction

Hepatitis B virus (HBV) is a partially double-stranded DNA virus. The persistence of HBV is attributable to its covalently closed circular DNA (cccDNA). cccDNA acts as a transcription template on a chromosome in the infected nucleus, encodes a pregenomic RNA (pgRNA) encapsulated by HBV polymerase, pgRNA is reverse transcribed by HBV DNA polymerase into partially double-stranded relaxed circular DNA (rcDNA)^1,2^. The viral genome has four overlapping open reading frames encoding the HBV core protein (HBc), the envelope protein, viral polymerase, reverse transcriptase, and regulatory HBX which is regarded as an oncoprotein. Studies have shown that HBeAg status is an indicator of viral replication fitness and disease prognosis^3^, and HBV-DNA levels have a predictive value for estimating hepatocellular carcinoma (HCC) risk and disease prognosis^4^. HBV is characterized by a narrow host range. HBV tends to replicate and infect hepatocytes, leading to acute and chronic hepatitis. Chronic HBV infection can progress to liver cirrhosis and HCC. According to the World Health Organization (WHO), 296 million people were infected with HBV in 2019, and an estimated 820,000 died of hepatitis B complications^5^. cccDNA and integrated HBV DNA is one of the barriers to HBV cure, current HBV therapeutics (such as pegylated-interferon-α (PEG-IFNα) and long-term nucleos(t)ide analogs (NUCs)) are not very effective^6^, the exploration of novel therapeutic approaches for HBV infection is necessary.

The RDA51 gene mediates DNA double-strand break repair by homologous recombination^7^ and encodes five isoforms (RAD51B, RAD51C, RAD51D, XRCC2, and XRCC3). RAD51C forms two distinct complexes, a tetramer (RAD51B/RAD51C/RAD51D/XRCC2; BCDX2) and a heterodimer (RAD51C/XRCC3; CX3), with other isoforms^8^. RDA51 is involved in the early stage of HR repair mediated by the CX3 complex and in the late stage by binding to Holliday Junction substrates, promoting branch migration and decomposition, thereby participating in the homologous recombination and repair of DNA^9,10^. RAD51C also recognizes and detects DNA damage signals and even transmits them to downstream pathways^11^, which plays a crucial role in various processes involved in DNA damage repair.

RAD51C is closely related to tumor development. It encodes a DNA repair protein that can contribute to the progression of genital cancer, and plays an important role in the DNA damage response (DDR)^12^ which is associated with the development and progression of human tumors. Mutations of this gene were first identified as increasing the susceptibility to breast and ovarian cancers^13,14^. RAD51C-deficient cancer cells are highly sensitive to Olaparib, a PARP inhibitor^12^, and RAD51C deficiency can be regarded as a biomarker predictive of the anti-tumor effects of Olaparib. In non-small cell lung cancer, increased expression of RAD51C can contribute to chemotherapy and/or radiotherapy resistance^15^. RAD51C expression is significantly increased in breast cancer and studies have also shown that clinicopathological features of early or familial gastric cancer are significantly correlated with the expression of RAD51C^16^. Early cDNA microarray results from our laboratory showed that RAD51C expression was significantly increased in HBV-infected HCC cells. This suggests that HBV infection can activate RAD51C expression and that activated RAD51C may be involved in the process of HBV infection leading to related diseases. So far, no relationship has been reported between viral infection and RAD51C expression. As such, we set out to investigate this link in the present study by utilizing an in vitro model of HBV infection.

## Materials and methods

### Plasmid construction

HBV-1.3 was produced in HepG2.2.15 cells (genotype D, subtype ayw, GenBank accession number U95551) and inserted into pBlue plasmids (Invitrogen) to obtain an expression plasmid carrying a 1.3-fold HBV genome sequence and producing infectious viral particles (p-Blue-HBV1.3). RAD51C expression plasmid pCAGGS-HA-RAD51C and pCDNA3.1( +)-3 × FLAG-HBE (HBC, HBP, HBX, HBs(L), HBs(S)) were constructed in our laboratory. The schematic diagram of plasmids was shown in supplementary Fig. S1. After the construction of sh-RAD51C expression plasmids, shRNA-expressing viruses were isolated by co-transfecting HEK293T cells with two packaging plasmids, psPAX.2 (Addgene, #12260) and pMD2.G (Addgene, #12259). Plasmid extraction kits were purchased from OMEGA, and the expression of all recombinant plasmids was validated by DNA sequencing and western blot analysis. Primers used for real-time fluorescent quantitative PCR and sh-RAD51C synthesis are shown in Table 1.

**Table 1:** Primers used for real-time fluorescent quantitative PCR and sh-RAD51C synthesis.

	Forward primer	Reverse primer
HBV pgRNA HBX sh-RAD51C	5′-TGGATTCGCACTCCTCCAGCTT-3′ 5′-GTCTGTGCCTTCTCATCTGCC-3′ 5′-CCGGGGGATTTAGAGATACTGTTGT-3′ 5′-CTCGAGACAACAGTATCTCTAAATCCC TTTTTG-3′	5′-GGGACCTGCCTCGTCGTCTA-3′ 5′-AGAGATGATTAGGCAGAGGTG-3′ 5′-AATTCAAAAAGGGATTTAGAGATAC-3′ 5′-TGTTGTCTCGAGACAACAGTATCTCTAAATCCC-3′
RAD51C GAPDH	5′-CCAGATCATATGAAGTGAATGGGA-3′ 5′-AAGGCTGTGGGCAAGG-3′	5′-TGGCCACATGAGATCAGCTTT-3′ 5′-TGGAGGAGTGGGTGTCG-3′

### Cell culture and establishment of the sh-RAD51C cell line

HepG2, HepG2.2.15, and Huh7 cells were cultured in DMEM cell culture media (Gibco) containing 10% fetal bovine serum (FBS) and 1% penicillin and streptomycin; HEK293T cells were cultured in DMEM cell culture fluid containing 10% FBS only. All cells were cultured at 37 °C and 5% CO_2_. The cells were transfected using Lipofectamine2000 (InvivoGen) according to manufacturer protocols (Lipofectamine 2000 2.5 µl with plasmids 1 µg). The supernatant of lentivirus isolated from HEK293T cells, which were co-transfected with sh-RAD51C, psPAX.2 and pMD2.G plasmids, and then HepG2 and Huh7 cells were infected at a ratio of culture fluid: virus liquid = 1:5. Polybrene (8 μg/ml) was added to increase the viral infection efficiency, and a RAD51C low-expressing cell line was established after puromycin (sellek, 2.5 μg/ml) selection.

### RNA extraction and RT-PCR

Total cellular RNA was extracted by using Trizol according to the instructions for the use of RNA extraction reagents which were purchased from CoWin Biosciences. Total RNA was reverse transcribed with oligonucleotides (DT), and glycerol-3-phosphate dehydrogenase (GAPDH) as a housekeeping reference gene.

### Enzyme-linked immunosorbent assay (ELISA)

HBeAg and HBsAg were detected by ELISA. 50 ul of the cell supernatant was added to a microplate pre-coated with human hepatitis B surface antibody (HBsAb) and hepatitis B e antibody (HBeAb) to fully bind to the well wall, and a drop of enzyme conjugate was added dropwise and then incubated at 37 °C for 1–2 h. The liquid in the wells was discarded, the plate was washed 5 times with phosphate-buffered saline (PBS), and the liquid was thoroughly dried with absorbent paper. Chromogen solutions A and B were added to each well at a time, 1 drop each. The plate was sealed and incubated in a 37 °C oven for 15 min, and the reaction was terminated by adding a stop buffer. Optical density (OD) was measured with a microplate reader at a wavelength of 450 nm and the results were analyzed.

### Animal experiments

The study was approved by the Ethics Committee of Renmin Hospital of Wuhan University (No. WDRY2020-K066), and all methods were carried out following relevant guidelines and regulations. This study was carried out in compliance with the ARRIVE guidelines 2.0. Nine male Babl/c mice were divided into three groups. Group 1 served as the control group and only received vector control. Group 2 was injected with pAAV-HBV plasmids into the mouse tail caudal veins. Group 2 was injected with pAAV-HBV and RAD51C plasmids into the mouse tail caudal veins. 7 days after injection, the mice were sacrificed by the cervical dislocation, and their blood and livers were collected for analysis.

### Co-immunoprecipitation (Co-IP)

The cell lysate was prepared by lysing transfected cells in 1 ml of lysis buffer (250 mM Tris–HCl, pH 7.4, 150 mM NaCl, 1 mM EDTA, 1% Triton X-100, and 10% glycerol). 500 μl of cell lysate and 2 μl of the corresponding antibody were added to a 1.5 ml centrifuge tube and incubated overnight on a shaker at 4 °C. The protein G magnetic beads (Invitrogen) were added and incubation was continued for another 2 h. After the beads were gently washed three times with 1 ml of cell lysate, 50 μl of 5 × Loading Buffer was added and pipetted to mix well, then boiled in water for 10 min and centrifuged at 12,000 rpm for 1 min at room temperature, and the supernatant was then used for western-blot detection. The original results of Co-IP were shown in supplementary Fig. S2 and Fig. S3.

### Immunofluorescence (IF) and confocal microscopy

Huh7 cells were fixed with 4% paraformaldehyde for 20–30 min at 4 °C, then washed three times with PBS, permeabilized with PBS containing 0.2% Triton X-100 (0.2% PBST) for 5 min, washed three times with PBS, and blocked with the PBS containing 10% BSA for 45 min at room temperature. The cells were rinsed three times with 0.2% PBST, then HA and Flag primary antibodies were added, and incubated overnight at 4 °C. The cells were rinsed three times with 0.2% PBST, then green fluorescent-labeled specific HA secondary antibodies and red fluorescent-labeled specific Flag secondary antibodies were added and incubated for 45–60 min at room temperature in the dark. The cells were rinsed three times with 0.2% PBST, then incubated with DAPI for 10 min at room temperature in the dark. Finally, the cells were observed by confocal fluorescence microscopy.

### Western blot

Cell lysates from transfected HepG2 and Huh7 cells were prepared, denatured, and electrophoresed using SDS-PAGE. After electrophoresis, proteins were transferred from the gel to a PVDF membrane. 5% skim milk powder in TBST was used to block at room temperature for 1 h. Then, RAD51C, GAPDH, and HBX primary antibodies were introduced and incubated overnight at 4 °C, and the corresponding HRP-labeled secondary antibodies were incubated at room temperature for 1 h before acquiring images.

### Statistical analysis

GraphPad Prism5 software was used to analyze data. The experiment was performed three independent times. Data were analyzed by *t* tests, and the results were considered significant at a p-value of < 0.05. Significance is indicated as follows: * = p < 0.05; ** = p < 0.01; *** = p < 0.001; or ns (not significant) = p ≥ 0.05.

## Results

### HBV infection promotes RAD51C expression in HCC cells

**Figure 1:**
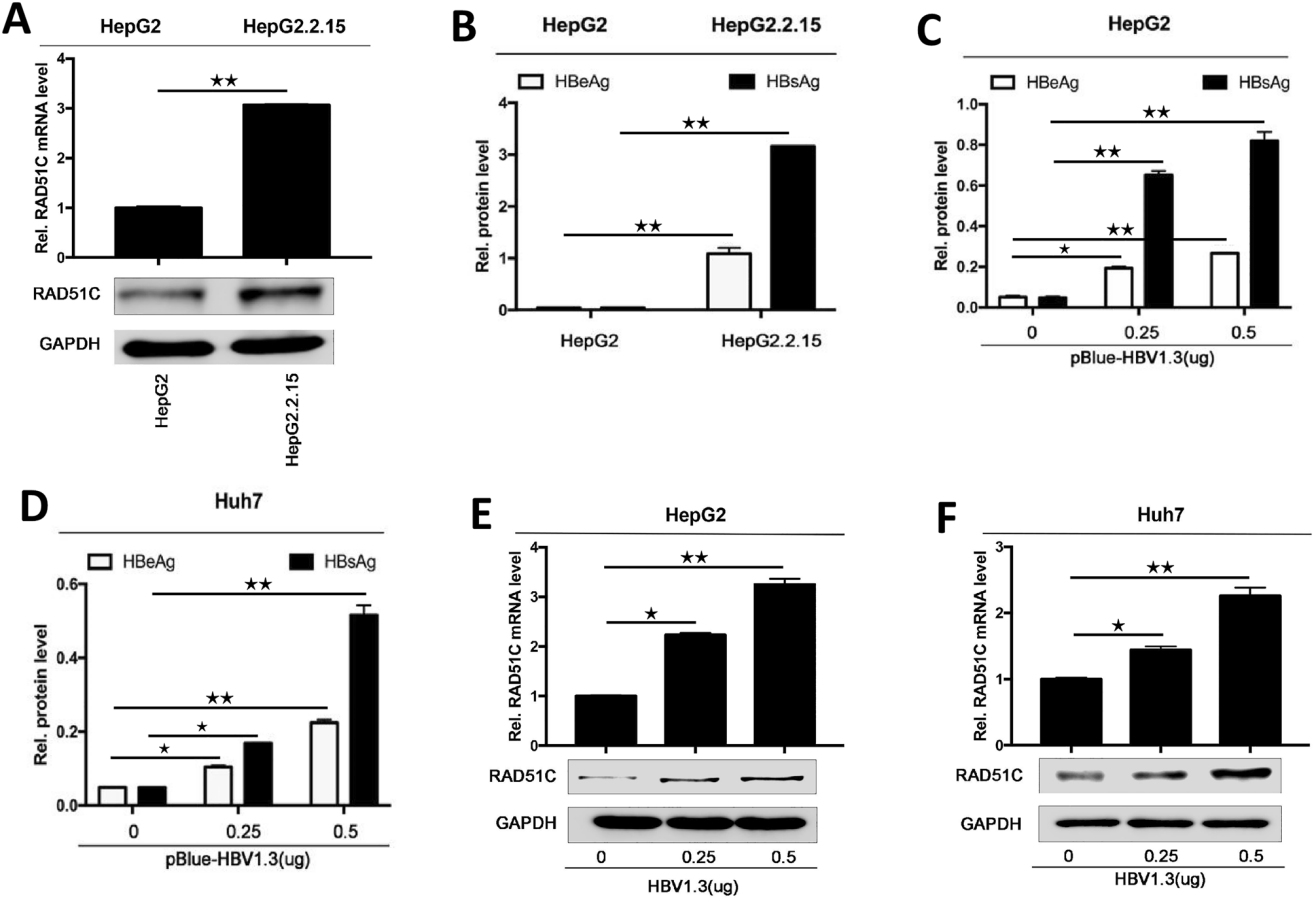
HBV activates RAD51C expression. (**A**) HepG2 cells and HepG2.2.15 cells stably expressing HBV1.3 were cultured for 48 h at 1 × 10^6^ cells per condition. Then, the supernatant and cells were collected. RAD51C protein was detected by western blot, and *RAD51C* mRNA was detected by RT-PCR. (**B**) Expression of HBeAg and HBsAg in the supernatant of HepG2 cells and HepG2.2.15 cells were detected by ELISA. pBlue-HBV1.3 was transfected into HepG2 cells (**C**,**E**) and Huh7 cells (**D**,**F**) and cultured for 48 h. (**C**,**D**) ELISA was used to test HBeAgs and HBsAgs in the cell supernatants, (**E**,**F**) RAD51C protein was detected by western blot and *RAD51C* mRNA was detected by RT-PCR. Data represent the mean ± SD, *p < 0.05, **p < 0.01 by an unpaired t-test.

HBV infection, RAD51C protein, and mRNA levels in HepG2 and HepG2.2.15 cells were detected by western blot and qRT-PCR. We found that with stable HBV expression, the RAD51C protein and mRNA levels in HepG2.2.15 cells were significantly higher than in the control HepG2 cells (Fig. 1A). We found that the viruses replicated successfully in HepG2.2.15 and HBV1.3 plasmid-transfected cells (Fig. 1B–D), and the RAD51C protein and mRNA levels increased with the increase in the gradient of HBV1.3 plasmids transfected into HepG2 and Huh7 cells (Fig. 1E and F).

### RAD51C promotes HBV replication in hepatocytes

**Figure 2:**
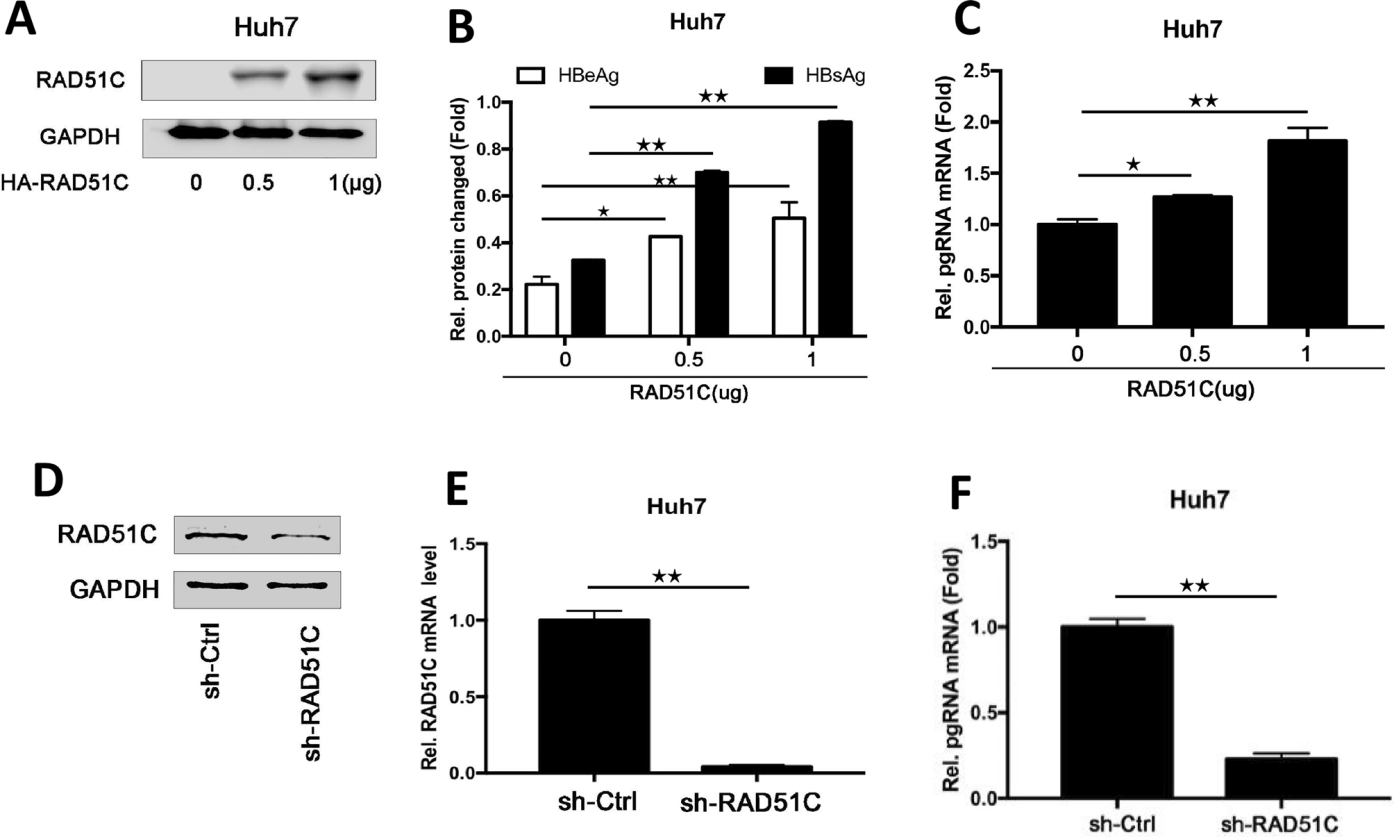
RAD51C promotes HBV replication in hepatocytes. pBlue-HBV1.3 and pCAGGS-RAD51C-HA of different concentrations were transfected into Huh7 cells and cultured for 48 h, supernatants and cells were collected. (**A**) The protein levels in pCAGGS-RAD51C-HA-transfected cells were detected by western blot, (**B**) HBeAgs and HbsAgs levels in the collected cell supernatants were detected by ELISA. (**C**) Total RNA was extracted from the cells, and HBV pgRNA levels in the cells were quantified by RT-PCR using *GAPDH* as a reference gene. After Huh7 cell infection by lentiviral packaging, pBlue-HBV1.3 was transfected into interfering and control cells and cultured for 72 h. RAD51C protein in transfected cells were detected by western blot (**D**), *RAD51C* mRNA and HBV pgRNA in the cells were detected by RT-PCR with GAPDH as a reference gene (**E**,**F**). Data represent the mean ± SD, *p < 0.05, **p < 0.01 by an unpaired t-test.

RAD51C and HBV1.3 plasmids were serially transfected into Huh7 cells (Fig. 2A). Supernatants and cells were collected, and RNA was extracted from one-tenth of the cells. The remaining cells were tested for transfection by western blot. ELISA kits were used to detect HBsAgs and HBeAgs levels in the cell supernatants. We found that overexpression of RAD51C promoted HBV antigen protein synthesis in HepG2 and Huh7 cells (Fig. 2B). We also found that in Huh7 cells, overexpression of RAD51C promoted HBV pgRNA expression (Fig. 2C), and HBV replication was in a dose-dependent manner. To further validate our conclusions, we also validated the effects of interfering with RAD51C expression on HBV replication. HBV was transfected into Huh7 cells interfering with RAD51C and into control cells; The efficiency of sh-RAD51C interference with RAD51C was detected by western blot and RT-PCR, showing that sh-RAD51C effectively down-regulated RAD51C expression (Fig. 2D and E). Then, HBV pgRNA was detected by RT-PCR (Fig. 2F). Our data showed that interfering with RAD51C can down-regulate the expression of HBV pgRNA and antigen protein synthesis compared with the control group. Interfering with RAD51C expression can inhibit HBV replication in hepatocellular Huh7 cells.

### RAD51C promotes HBV replication in mice

**Figure 3:**
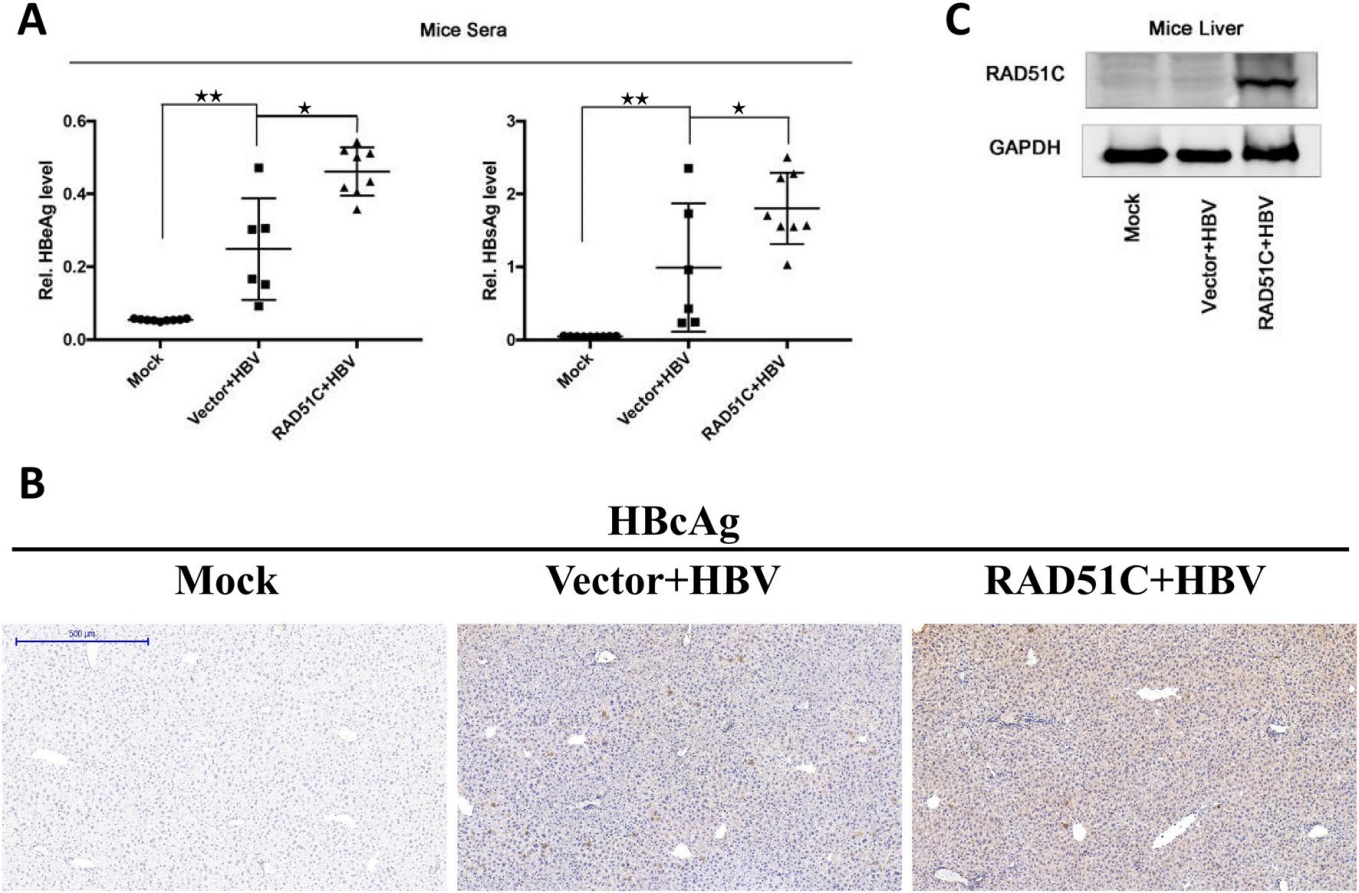
RAD51C promoted HBV infection in mice. (**A**) The effects of RAD51C on HBV replication were investigated in 4-week-old Babl/c mice. HBeAg and HBsAg blood levels detected by ELISA are shown, each point in the figure representing one animal. (**B**) Liver tissues were sectioned and stained with HBcAg antibodies for immunohistochemistry. (**C**) RAD51C plasmid protein levels which were injected into the caudal veins of the mice under high pressure were detected by western blot. Data represent the mean ± SD, *p < 0.05, **p < 0.01 by an unpaired t-test. Scale bar = 500 µm.

To further demonstrate that RAD51C proteins can also promote HBV replication in vivo, we used a Babl/c mouse model. A pAAV-HBV and RAD51C plasmid, or vector control were injected into the mouse tail caudal veins under high pressure leading the plasmids to be transmitted into the heart via the high-pressure blood to induce an acute HBV infection in the mice. 7 days after infection, the mice were sacrificed, and their blood and livers were collected for analysis. The liver tissue was used for immunohistochemistry analysis. Their blood was centrifuged with an anticoagulant, and the supernatant was diluted tenfold. Then, we used ELISA to quantify the levels of HBsAg and HBeAg in the blood (Fig. 3A). We found that HBsAg and HBeAg blood levels were higher in mice injected with RAD51C plasmids than in the vector control. The liver tissue of HBV-infected mice underwent immunohistochemistry with specific antibodies to detect the levels of HBV core proteins and RAD51C proteins in the hepatocytes (Fig. 3B). We found that the level of RAD51C protein was significantly higher in the liver of mice injected with RAD51C plasmids compared to the vector control group (Fig. 3C).

### RAD51C protein interacts with HBX proteins

**Figure 4:**
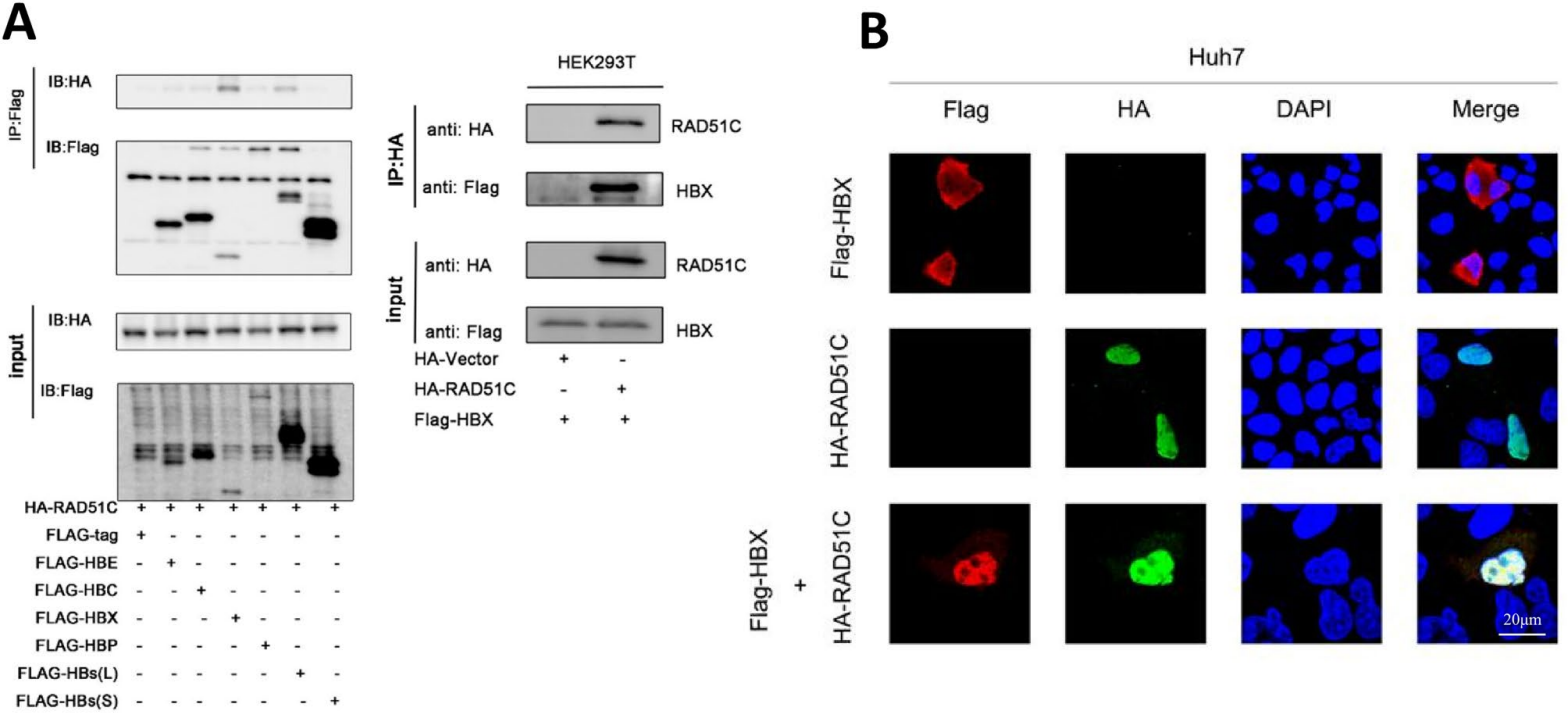
RAD51C proteins in the nucleus interacted with HBX proteins. (**A**) HEK293T cells were co-transfected with pFlag-tag, pFlag-HBE, pFlag-HBC, pFlag-HBX, pFlag-HBP, pFlag-HBs(L), pFlag-HBs(S), and HA-RAD51C, then cultured for 48 h, collected, and prepared as a whole-cell lysate for Co-IP. Co-IP reactants were detected by western blot. (**B**) HA-RAD51C and Flag-HBX plasmids were co-transfected into Huh7 cells; nuclei were stained with green fluorescent-labeled specific HA secondary antibodies, red fluorescent-labeled specific Flag secondary antibodies, and DAPI to observe localization by confocal fluorescence microscopy. Scale bar = 20 µm.

Flag-tagged HBE, HBC, HBX, HBP, HBs(L), and HBs(s) plasmids and HA-tagged RAD51C plasmids were co-transfected in HEK293T cells. Co-IP was performed at the end of the culture. We found that RAD51C and HBX proteins interacted with each other regardless of forward and reverse pull-down (Fig. 4A).

Huh7 cells were cultured in a confocal dish, then transfected with Flag-HBX alone, HA-RAD51C alone, and co-transferred with Flag-HBX and HA-RAD51C. Nuclei were stained with red fluorescent-labeled specific Flag secondary antibodies, green fluorescent-labeled specific HA secondary antibodies, and DAPI. The subcellular location of the proteins and the spatial location of the interaction between RAD51C and HBX were observed by confocal fluorescence microscopy. In the case of a single transfection, Flag-HBX was mainly located in the cytoplasm and HA-RAD51C was in the nucleus. In the case of co-transfection, Flag-HBX was mainly located in the nucleus. HBX and RAD51C co-localized in the nucleus (Fig. 4B). These findings demonstrate that RAD51C and HBX proteins co-localize in the nucleus, and the RAD51C protein can cause the migration of HBX proteins from the cytoplasm to the nucleus.

## Discussion

Chronic HBV infection remains an important public health challenge. As a major cause of chronic HBV, liver cirrhosis, and liver cancer, the pathogenesis of chronic HBV infection remains incompletely understood. cccDNA and integrated HBV DNA are barriers to HBV cure, current HBV efficacy using PEG-IFNα and NUCs is unsatisfactory; significant efficacy mainly appears in patients with low HBsAg levels^6^; and exploring the replication mechanism of the HBV is crucial for finding new treatments. The main purpose of our study was to investigate the relationship between HBV infection and RAD51C. Our study findings provide insights into HBV pathogenesis and potentially inform the identification of effective methods to prevent and treat HBV-related diseases.

RAD51C is involved in homologous recombination and repair of DNA, playing a critical role in maintaining genome stability^17–19^. RAD51C is closely related to tumors. RAD51C encodes the DNA repair protein that may contribute to the progression of genital cancer and play an important role in the DNA damage response (DDR) ^20^, which is associated with the development and progression of human tumors. RAD51C has been investigated in many previous studies, mainly focusing on ovarian cancer, breast cancer, and other neoplastic disorders^21^. However, the role of RAD51C in regulating HBV infection has not been reported until now. We demonstrate for the first time that there is a correlation between RAD51C expression and HBV replication. RAD51C mRNA and protein were significantly up-regulated in HBV-infected HCC cells. Therefore, there may be a potential regulatory relationship between RAD51C expression and HBV replication, which plays a role in the development of HBV-related diseases.

On this basis, we performed an in-depth study of the RAD51C protein’s regulation of HBV replication. First, we confirmed that HBV infection activated RAD51C mRNA and protein in the transfected HCC cells (Fig. 1). Second, we explored the effects of activated RAD51C expression on HBV replication. Relying on overexpression and interference tests in Huh7 cells, we confirmed that RAD51C promoted the expression of HBV pgRNA, HBsAg, and HBeAg in a dose-dependent manner. In addition, RAD51C was shown to promote HBV replication in mice in vivo (Fig. 3), revealing a positive feedback regulatory mechanism between RAD51C and HBV.

Finally, we further explored the interaction between RAD51C and HBV elements. We first screened HBX proteins that interacted with RAD51C (Fig. 4). We then found that RAD51C and HBX proteins co-localized in the nucleus and interacted as demonstrated by Co-IP, and that RAD51C protein caused the migration of HBX protein from the cytoplasm to the nucleus. HBx protein binds the promoter region of DLEU2, which enhances the transcription of DLEU2 in infected hepatocytes. Then, the co-recruitment of HBx and DLEU2 on the cccDNA boosts the transcription and replication of HBV^22^.

HBX, encoded by a highly conserved X open reading frame (ORF), is a protein composed of 154 amino acids. In HBV-infected hepatocytes, HBX proteins were highly expressed in the cytoplasm and sparsely expressed in the nucleus. HBX in the cytoplasm co-localized with mitochondria^22–26^ and stimulated HBV replication by localizing in the nucleus^27^. In vitro studies have shown that HBX induces apoptosis by promoting the production of reactive oxygen species (ROS), activating caspase 8, removing mitochondrial membrane potential, and releasing cytochrome c while inhibiting p53^28^, Fas, TNF^29^, and TGFb-induced apoptosis. The seemingly contradictory effects of HBX on regulating apoptosis suggest a complex and important role for this protein in hepatocarcinogenesis. Meanwhile, the C-terminal-truncated HBX was also involved in hepatocarcinogenesis. Therefore, we speculate that HBV may activate the expression of RAD51C, increased expression of the RAD51C causes the aggregation of HBX in the nucleus, which accelerates the progression of HBV-related diseases. The molecular mechanism of the interaction between RAD51C and HBX still need to be elucidated in the following study.

In summary, we conclude that HBV up-regulates RAD51C, which itself promotes HBV replication and exerts biological effects through direct interaction with the HBX protein, laying the foundation for revealing the role of RAD51C in HBV pathogenesis and carcinogenesis.

### Supplementary Information

Supplementary Figures.

## Data Availability

The datasets generated and analysed during the current study are available in the http://www.ncbi.nlm.nih.gov/genbank/. Accession number U95551.
