# Supplementary material for: HBV promotes its replication by up-regulating RAD51C gene expression
Source: Sci Rep. 2024 Jan 31;14:2607. doi: 10.1038/s41598-024-53047-7 (PMC10831117; doi:10.1038/s41598-024-53047-7)
Supplement: Supplementary file 1 — Supplementary Figures. [file 41598_2024_53047_MOESM1_ESM.pdf]

HBV Promotes Its Replication by Up-regulating RAD51C Gene Expression

Ting-wei Peng[1#], Qing-feng Ma[2#], Jie Li[3#], Xue Wang[1], Cong-hui Zhang[1], Junwen Ma[2], Jun-yi Li[2], Wei Wang[4*], Cheng-liang Zhu[5*], Xing-hui Liu[1*]

1. Department of Clinical Laboratory, Shanghai Gongli Hospital, the Second Military Medical University, Shanghai 200135, China

2. Department of Clinical Laboratory, Liyuan Hospital of Tongji Medical College, Huazhong University of Science and Technology, Wuhan 430077, China

3. China Medical Tribune, Beijing 100009, China

4. Department of Clinical Laboratory, Wuhan Fourth Hospital of Tongji Medical College, Huazhong University of Science and Technology, Wuhan 430034, China

5. Department of Clinical Laboratory, Renmin Hospital of Wuhan University, Wuhan 430060, China

[#]The authors contributed equally to this work

[*]Corresponding authors

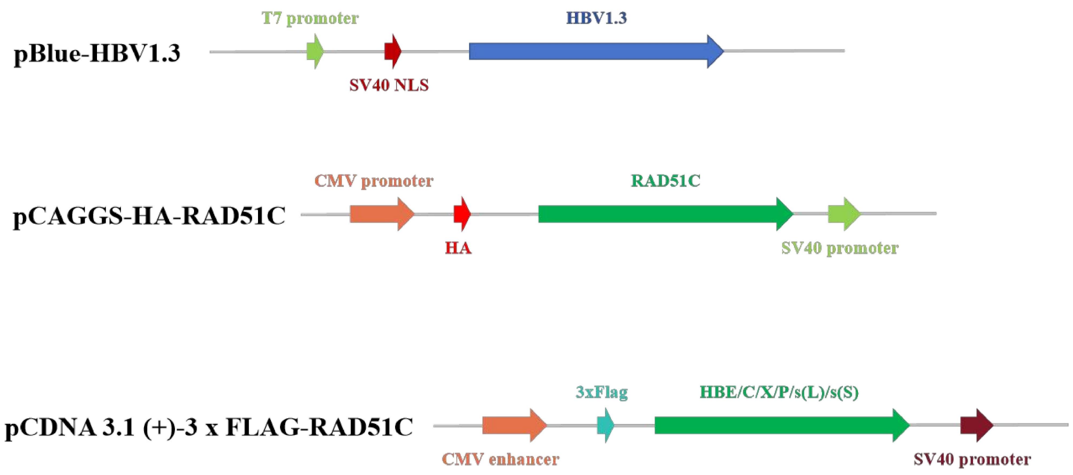

Supplementary Figure S1. The schematic diagram of plasmids (pBlue-HBV-1.3, pCAGGS-HA-RAD51C, and pCDNA3.1(+)-3x FLAG-RAD51C.

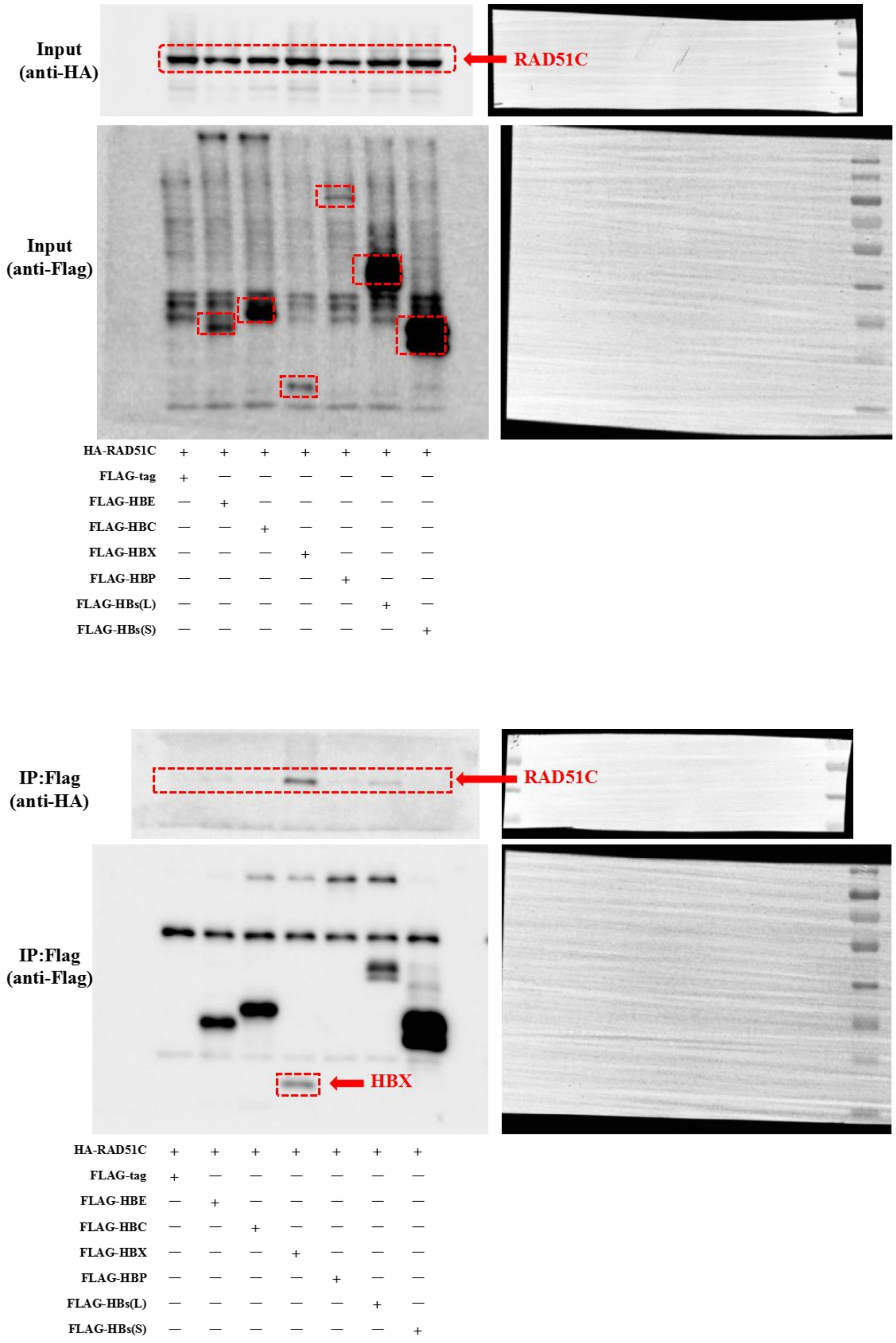

Supplementary Figure S2. Co-immunoprecipitation (HA-RAD51C pulls down Flag-HBX). HEK293T cells were co-transfected with pFlag-tag, pFlag-HBE, pFlag-HBC, pFlag-HBX, pFlag-HBP, pFlag-HBs(L), pFlag-HBs(S), and HA-RAD51C, then cultured for 48 h, collected, and

prepared as a whole-cell lysate for Co-IP.

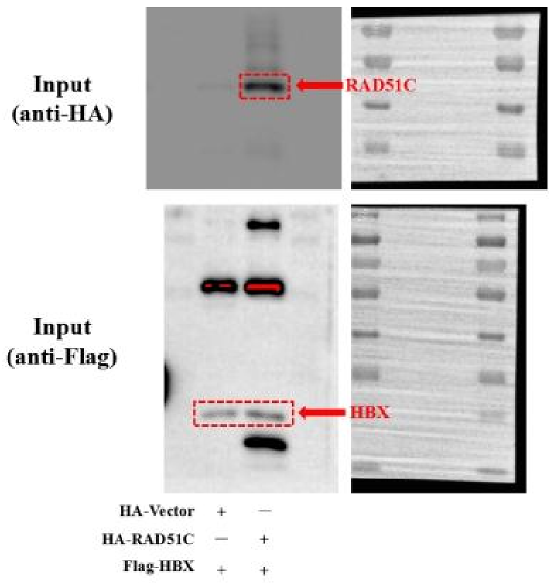

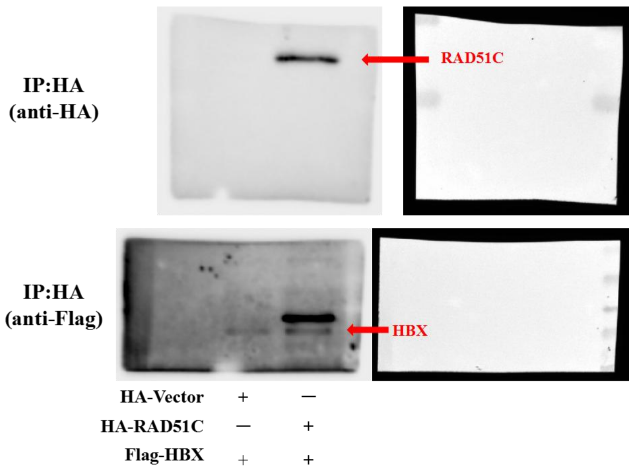

Supplementary Figure S3. Co-immunoprecipitation (Flag-HBX pulls down HA-RAD51C). HEK293T cells were co-transfected with pFlag-tag, pFlag-HBE, pFlag-HBC, pFlag-HBX, pFlag-HBP, pFlag-HBs(S), pFlag-HBs(L), and HA-RAD51C, then cultured for 48 h, collected, and prepared as a whole-cell lysate for Co-IP.
